# Paediatric T-cell lymphoma of the appendix: a case report

**DOI:** 10.1186/1746-1596-8-2

**Published:** 2013-01-09

**Authors:** Yoshifumi Matsushita, Morishige Takeshita

**Affiliations:** 1 Laboratory of Pathology, Chidoribashi Hospital, 5-18-1 Chiyo, Hakata-ku, Fukuoka, 812-8633, Japan; 2 Department of Pathology, Faculty of Medicine, Fukuoka University, Nanakuma 7-45-1, Jonan-ku, Fukuoka, 814-0180, Japan

**Keywords:** T-cell lymphoma, Appendix, Child

## Abstract

A 7-year-old boy with no history of malnutrition or diarrhoea complained of acute abdominal pain, was diagnosed with acute appendicitis, and underwent appendectomy. Histologically, a diffuse infiltrate of large atypical lymphoid cells was found in the entire appendiceal wall. Immunohistochemical examination revealed that the tumour cells expressed T-cell receptor (TCR)-βF1, CD3, CD4, CD25, cytotoxic-related protein TIA1 and granzyme-B, but were negative for CD8, Foxp3, CD20, CD30 and CD56. Polymerase chain reaction (PCR) revealed clonal bands of TCR-γ gene products in the tumour tissue. No anti-cytomegalovirus antibody-positive cells were detected. In situ hybridization revealed no nuclear signals of Epstein-Barr virus (EBV)-encoded RNA. *Helicobacter pylori* infection was detected in tumour tissue by anti-East Asian cytotoxin-associated gene (Cag) A antibody and PCR using its specific primers. The patient received chemotherapy and has remained in remission for 2 years. To the best of our knowledge, only two cases of appendiceal T-cell non-Hodgkin lymphoma (NHL) have been reported, both in elderly patients. We believe that this is the first reported case of childhood CD4- and TIA1-positive cytotoxic T (Th1)-cell NHL in the appendix or gastrointestinal tract. *Helicobacter pylori* infection might be an initiator of atypical cytotoxic T-cell proliferation.

**Virtual Slides:**

The virtual slide(s) for this article can be found here: http://www.diagnosticpathology.diagnomx.eu/vs/1302380563830412.

## Background

The specification of non-Hodgkin lymphoma (NHL) in the “WHO Classification 2008” is based on recent progress in immunohistochemical and genetic analysis and clinical findings, including prognostic data [1]. Among extranodal NHLs, the alimentary tract is the most frequently affected site [1,2]. In the small intestine, diffuse large B-cell NHL (DLBCL) is the most common subtype of lymphoma, followed by mucosa-associated lymphoid tissue (MALT) lymphoma in the elderly, while Burkitt’s lymphoma is the predominant childhood NHL. Appendiceal involvement is extremely rare, constituting less than 1% of all small- and large-intestinal NHLs [2,3]. About 50 cases of appendiceal NHL have been previously reported, but immunohistochemical and genetic examinations have been performed in only a limited number of recent cases, including two T/natural killer (NK)-cell NHL cases [2-5]. Among intestinal T/NK-cell NHLs, enteropathy-associated T-cell lymphoma (EATL) has been frequently reported in the elderly [1,6-8]. Type I EATL is a CD4- and CD8-negative and CD30-positive large-cell lymphoma associated with coeliac disease, and is seen in northern Europe and the United States. Type II EATL is a CD56-positive and CD8-positive or -negative medium-sized lymphoma that is less strongly associated with coeliac disease. There are several reports of nasal-type NK-cell lymphomas with Epstein-Barr virus (EBV) infection in intestine and colon, which are usually encountered in the elderly and rarely in children [7,9]. The present case is a CD3-, CD4- and TIA1-positive and CD30-negative (Th1) large-cell lymphoma without EBV infection. CD4-positive T/NK-cell lymphoma has occasionally been reported in the stomach, but is rarely seen in the intestine [10,11]. The intestinal bacterial florae, *Helicobacter pylori* and *Campylobacter jejuni,* may be initiators of abnormal lymphocytic proliferation in the stomach and intestine [12,13]. Here, we present what is, to the best of our knowledge, the first report of a childhood case of appendiceal CD4-positive T-cell NHL and discuss the influence of *H. pylori* infection.

## Case presentation

### Clinical history

A 7-year-old boy was referred to our hospital with complaints of abdominal discomfort and high fever. Four days before admission, the patient complained of uneasiness and sneezing and was afebrile. On the following day, the patient complained of increasing abdominal pain and appetite loss. One day before admission, the symptoms worsened and the patient’s temperature rose to 39.1°C. There was no history of recurrent diarrhoea, malnutrition or failure to thrive. On admission, the white blood cell count remained within normal limits, but C-reactive protein was elevated to a concentration of 3.6 mg/dL. An abdominal ultrasound revealed a mildly swollen appendix. In addition, a few mildly swollen lymph nodes, up to 10 mm in diameter, were seen in the mesentery. A diagnosis of acute appendicitis was made and appendectomy was performed on the second day of hospitalisation.

## Material and methods

### Immunohistochemistry

The antibodies used in this study were as follows: TCR-βF1 (Endogen, Rockford, IL, USA); CD3, CD5, CD7, CD8, CD25, CD56, CD57, and terminal deoxynucleotidyl transferase (TdT) (Novocastra, Newcastle, UK); CD4 (MBL, Nagoya, Japan); Foxp3 (e-Bioscience, San Diego, CA, USA); TIA-1 (Immunotech, Marseille, France); Granzyme B (Chemicon, Temecula, CA, USA); CD20 (Nichirei, Tokyo, Japan); and CD79a, CD30, CD15, anaplastic lymphoma kinase (ALK), myeloperoxidase, epithelial membrane antigen (EMA), AE1/AE3 and anti-cytomegalovirus (CMV) antibody (Dako Cytomation, Glostrup, Denmark). Anti-East Asian *H. pylori* CagA antibody was kindly provided by Dr. T. Uchida, Department of Molecular Medicine, Oita University, Japan [14].

### In situ hybridisation for detection of Epstein-Barr virus (EBV)-encoded RNAs

Tissue sections were digested with proteinase K and incubated in a solution of 50% formamide containing digoxigenin/biotin-labelled EBV-encoded RNA (EBER) oligonucleotide probes (Dako Cytomation). A peroxidase-conjugated anti-FITC antibody was applied to the sections to detect the hybridized probes.

### Polymerase chain reaction (PCR) for T-cell receptor (TCR)-γ and *H. pylori*-associated genes

For evaluations of genes associated with TCR-γ and *H. pylori*, DNA was extracted from paraffin-embedded tumour sections. TCR-γ gene analysis was performed according to the BIOMED II PCR method [15].

The East Asian-type CagA genes were detected using primer sets CAGJF/CAGTR and CAGTF/CAGJR, which yielded 222- and 293- to 299-bp products, respectively [16].

## Results

### Pathology

**Figure 1:**
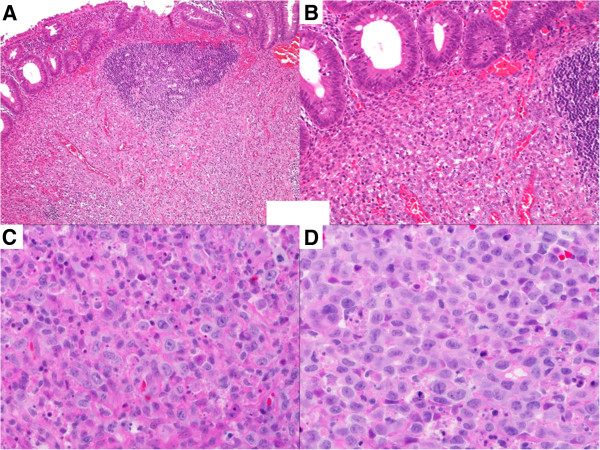
**Histological features of the appendix. (A)** Atypical lymphoid cells and preserved lymph follicle are in submucosal layer (H&E stain, ×100). (**B)** Intraepithelial lymphocytes are not increased, and abnormal tumour cell invasion is not prominent in the mucosal layer (H&E stain, ×200). Large atypical lymphocytes with small distinct nuclei diffusely infiltrate the (**C**) submucosal layer and (**D**) eroded lesion. Many reactive histiocytes are seen, mainly in (**C**) (H&E stain, ×400).

Macroscopically, the excised appendix was 4.0 × 0.8 cm in size, with an eroded mucosal layer and a haemorrhage on the serosal surface. However, there was no evidence of tumour formation or perforation. Microscopically, the mucosal layer appeared severely eroded with remnants of mucosal tissue. Intraepithelial lymphocytes (IELs) were not increased in number and lymphoid follicles with germinal centres were preserved (Figure 1A). Atypical large lymphoid cells with round nuclei were found mainly in the submucosal and muscle layers (Figure 1B). Extensive and diffuse invasion by large atypical lymphocytes can be seen in the eroded lesion. A severe histiocytic reaction involving many phagocytic macrophages was noted in the area of tumour cell invasion (Figure 1C, 1D). No definite granuloma formation was detected in the sections examined. Based on these findings, malignant lymphoma, rather than epithelial tumour, was highly suspected. The ileal tissue excised during appendectomy revealed oedematous mucosa and no infiltrating atypical cells.

### Immunohistochemistry and genetic analysis

**Figure 2:**
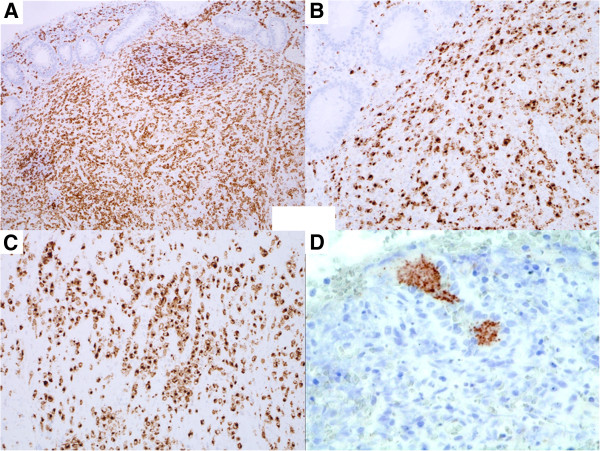
**Immunohistological findings in the appendix. (A**) CD3-positive tumour cells distributed mainly in the submucosal and muscle layers (×100). Infiltrating atypical large lymphoid cells are positive for (**B**) CD4 and (**C**) TIA1 (×200). TIA1-positive lymphoma cells invade the muscle layer. (**D**) Rod-like substances in the foveolar pit are positive for anti-East Asian type *H. pylori* CagA antibody (×400).

**Figure 3:**
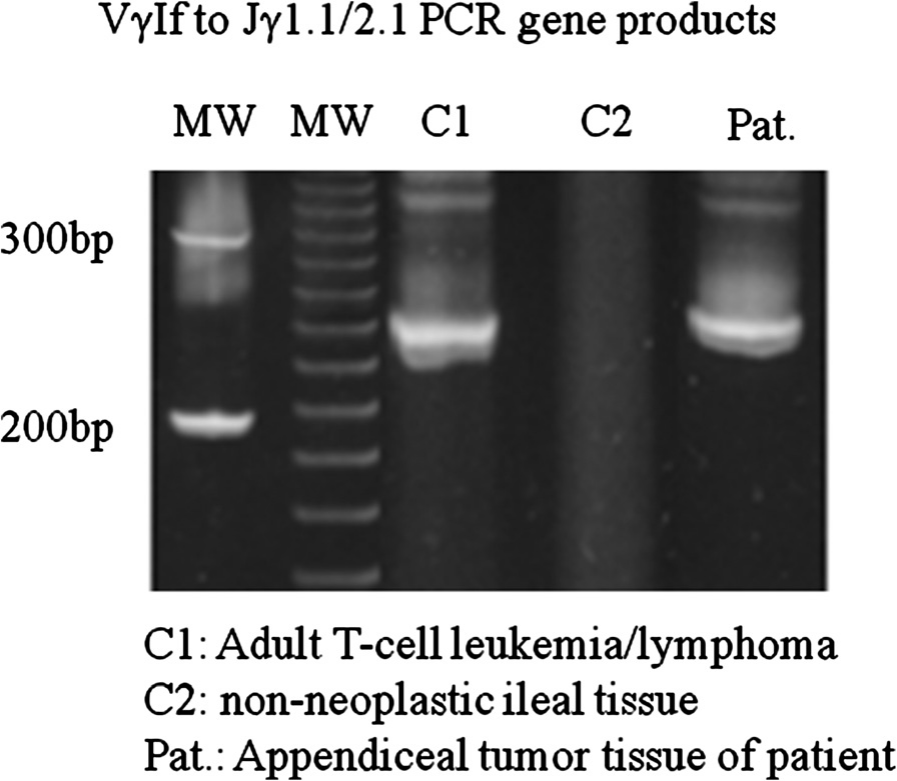
**Detection of TCR-Vγ1f to -Jγ1.1/2.1 gene products.** C1: nodal adult T-cell leukaemia/lymphoma (positive control); C2: non-neoplastic intestinal specimen (negative control); Pat.: patient sample. The 230 bp clonal band (TCR-Vγ to **Jγ**) is identified in lane C1 and in the patient sample.

**Figure 4:**
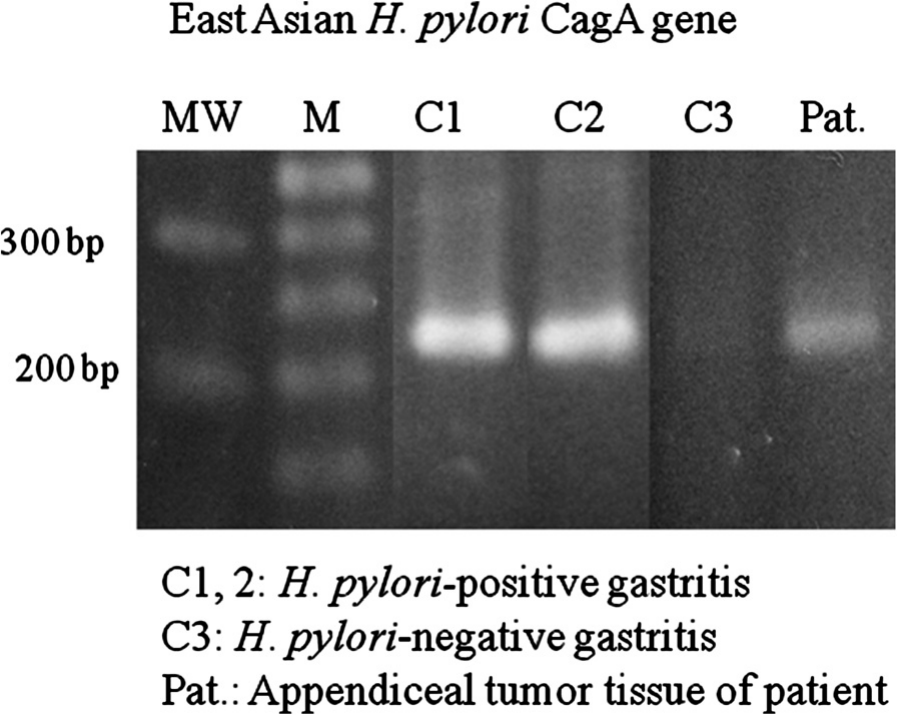
**Detection of the CagA gene of the East Asian type *H. pylori*.** The CagA gene (222 bp clonal bands) detected in DNA extracted from tissue specimens using primer set CAGJF/CAGTR. C1 and C2: *H. pylori***-**positive gastritis cases. C3: *H. pylori***-**negative gastritis case. Pat.: patient sample.

Immunohistochemical analysis revealed that the atypical tumour cells were positive for CD3 (Figure 2A), TCR-βF1, CD4 (Figure 2B), CD5, CD7, CD25, cytotoxicity-related protein TIA-1 (Figure 2C) and granzyme B, but were negative for TCR-CγM1, CD8, Foxp3, CD15, CD20, CD79a, CD30, ALK-1, CD56, CD57, TdT, myeloperoxidase, lysozyme, EMA and cytokeratin AE1/AE3. The MIB-1 (Ki-67) labelling index was greater than 80%. CD3-positive large lymphoid cells diffusely infiltrated into the submucosal and muscle layers, and partly infiltrated into the mucosal layer. TCR-γ gene analysis identified a 230-bp clonal band of TCR-γ gene tube A by the BIOMED II method for PCR (Figure 3). Based on these results, we determined that this lesion in the appendix was a lymphoma consisting of a diffuse infiltration of primary CD4- and TIA-1-positive cytotoxic T (Th1) cells. No anti-CMV-positive mononuclear cells were detected in the tissue. EBV-encoded RNA (EBER)-positive nuclear signals were not detected in tumour tissue by in situ hybridization. Anti-East Asian CagA-positive bacterial-like substances were detected in the epithelial pits (Figure 2D) and in the infiltrating macrophages among the tumour cells. By PCR, clonal bands (222 bp and 293 to 299 bp) of the East Asian CagA genes were detected by two probes (Figure 4).

### Follow-up clinical data and history

Serum interleukin-2 receptor (sIL-2R) was 2,451 U/mL in a pre-surgical blood sample, which decreased to 1,089 U/mL 1 week after appendectomy. Anti-EBV antibodies had not increased, and anti-human T-lymphotropic virus-1 (HTLV-1) antibody was negative. No evaluation of serum anti-*H. pylori* antibodies was performed. The patient was transferred to another hospital with a paediatric haematology facility for intensive chemotherapy, and has been in remission for 2 years.

## Discussion

A study by Gustafsson et al. of 2,757 appendiceal tumours included 47 NHLs; immunological studies were performed in 11 cases and all were DLBCL. Several cases of appendiceal MALT-type lymphoma, mantle cell lymphoma and Burkitt’s lymphoma have been reported [17-19]. The present case was diagnosed as CD3-, CD4-, CD5-, CD7-, CD25- and TIA1-positive cytotoxic T-cell NHL. In childhood, ALK-positive anaplastic large cell lymphoma is a major type of CD4- and TIA1-positive cytotoxic T/NK-cell lymphoma. In the present case, this diagnosis was unlikely, because there was no expression of CD30, ALK and EMA [1]. Most intestinal T/NK-cell NHL are EATL, especially in the jejunum. Patients with EATL usually complain of diarrhoea, malnutrition and abdominal pain [1,6]. Type I EATL is a CD4- and CD8-negative and CD30-positive large-cell NHL. Type II EATL is a CD4-negative, CD8-positive or -negative and CD56-positive medium-sized NHL [1,8,11]. In addition, EATL expresses CD7 and TIA1, and is negative for CD4, CD5 and CD25. The current patient had no history of recurrent diarrhoea and malnutrition, which are both frequently found in cases of coeliac and Crohn’s diseases. Intraepithelial lymphocytes (IELs), which are typical for EATLs, were not found in the present case. Weiss et al. [20] reported on a 6-year-old patient with NK cell-like T-cell lymphoma restricted to the jejunum; the tumour cells were positive for CD3 and CD56 and negative for CD4, CD8 and CD30, and there was no EBV infection, similar to type II EATL. Considered together, these findings suggest that our patient’s lesion had clinicopathological and phenotypic characteristics different from those of EATL.

Primary T-cell NHL involving the appendix has previously been reported in two elderly patients. Kitamura et al. [4] reported on a case of T/NK-cell NHL in an 84-year-old male. In their study, tumour cells expressed CD3, CD8 and granzyme-B, but were negative for EBV infection. Another case was a 45-year-old male who had received a renal transplant 17 years earlier and had subsequently developed CD56-positive nasal-type EBV-positive large T/NK-cell lymphoma [5]. The two previously reported cases of appendiceal T-cell NHL occurred in adults. Therefore, the authors believe that this is the first reported case of childhood CD4- and TIA-1-positive cytotoxic T-cell lymphoma in the appendix, or, indeed, in the entire gastrointestinal tract.

This patient received cytotoxic treatment and has been in remission for 2 years. Chuang et al. [7] evaluated 24 cases of primary T-cell NHL and 6 cases of NK-cell NHL in the gastrointestinal tract [1]. According to their report, using univariate and multivariate COX proportional hazard regression analysis, NK-cell lineage was associated with poor prognosis. EBV infection plays an important role in the progression of various NHLs [21]. We speculate that the early clinical stage and EBV-free status of the current patient predicted better prognosis. However, this is a single case and the follow-up period was limited. Identification of additional cases of intestinal T/NK-cell NHL and long-term follow-up is necessary in order to fully understand the clinical features of appendiceal T/NK-cell NHL.

In Japan, gastric carcinoma and MALT-type lymphoma have higher incidences compared with those occurring in other regions of the world [22]. It was strongly suggested that the East Asian CagA gene and protein have a great influence on the tumourigenesis of these two disorders [14,15]. Kiriya et al. [23] demonstrated that the T-cell reaction against the captured, round-shaped *H. pylori* seen in dendritic cells of Peyer’s patches in the small intestine plays a critical role in *H. pylori* gastritis. CD4-positive T cells, including Th1 and regulatory T cells, are distributed in the gastric mucosa in *H. pylori* infection [24], and cases of primary CD4-, CD5-, CD25- and TIA1-positive cytotoxic T-cell lymphoma have been reported in the stomach [10]. Among CD4-positive T cells, neoplastic cells of the present case had phenotypic findings regarding TIA1 expression similar to those of Th1 effector cells [25]. *Helicobacter pylori* infection might play a role in abnormal proliferation of CD4-positive cytotoxic T (Th1) cells. However, although Küpeli et al. [26] in Turkey reported that 3 of 15 cases (20%) of childhood systemic NHL had serological *H. pylori* infection and that 2 cases were T-cell type ALCL, they suggested that *H. pylori* infection was not an agent responsible for lymphomagenesis.

## Conclusion

We present a rare paediatric case of appendiceal CD3-, CD4- and TIA1-positive cytotoxic T (Th1)-cell lymphoma. Further studies are necessary to examine the relationships between *H. pylori* infection, including the Asian variety, and NHL.

## Competing interests

The authors declare that they have no conflicts of interest.

## Authors’ contributions

YM carried out initial pathological diagnosis of this case. YM and MT participated in the sequence alignment and drafted the manuscript. Both authors read and approved the final manuscript.
